# Oral intake of *Lactobacillus pentosus *strain b240 accelerates salivary immunoglobulin A secretion in the elderly: A randomized, placebo-controlled, double-blind trial

**DOI:** 10.1186/1742-4933-7-11

**Published:** 2010-08-26

**Authors:** Yoshifumi Kotani, Shoji Shinkai, Hiroshi Okamatsu, Masamichi Toba, Kishiko Ogawa, Hiroto Yoshida, Taro Fukaya, Yoshinori Fujiwara, Paulo HM Chaves, Keiji Kakumoto, Noriyuki Kohda

**Affiliations:** 1 Otsu Nutraceuticals Research Institute, Otsuka Pharmaceutical Co., Ltd., Shiga, Japan; 2 Research Team for Social Participation and Community Health, Tokyo Metropolitan Institute of Gerontology, Tokyo, Japan; 3 Center on Ageing and Health, Division of Geriatrics, Johns Hopkins Medical Institutions, Baltimore, Maryland, USA; 4 Information Management Office, Drug Safety Research Center, Tokushima Research Institute, Otsuka Pharmaceutical, Co., Ltd., Tokushima, Japan

## Abstract

**Background:**

Immunoglobulin A (IgA) secretion in saliva decreases with age and may be the cause of increased vulnerability of the elderly to respiratory infections. The effect of oral intake of lactic acid bacteria on salivary secretory IgA (SIgA) in the elderly has not been reported. The objective of this study was to demonstrate the acceleration of salivary SIgA secretion by oral intake of *Lactobacillus pentosus *strain b240 (b240) in the elderly.

**Results:**

A total of 80 healthy elderly individuals were randomly allocated to either an intervention (i.e., b240) or a control (i.e., placebo) group. The elderly individuals in the b240 group were given a sterile water beverage (125 mL) containing heat-killed b240 (4 × 10^9 ^cells), while those in the placebo group were given only a sterile water beverage (125 mL); both groups received their respective beverages once daily for 12 weeks. Saliva was collected before initiation of the study and every 2 weeks thereafter. Saliva flow rate and SIgA concentration were determined, and the SIgA secretion rate was calculated. The mean salivary SIgA secretion rate in the b240 group steadily increased until week 4 (exhibiting a 20% elevation relative to that at week 0), and then remained stable until week 12. Changes in SIgA secretion rate over the intervention period were significantly greater in the b240 group than in the placebo group. The treatment groups exhibited no significant differences in adverse events.

**Conclusions:**

Oral intake of *L. pentosus *strain b240 for 12 weeks significantly accelerated salivary SIgA secretion, thereby indicating its potential utility in the improvement of mucosal immunity and resistance against infection in the elderly.

## Background

The human body has various defense mechanisms against pathogenic microorganisms. The mucosal membranes covering the oral cavity, gastrointestinal, respiratory, and genitourinary tracts are continuously exposed to pathogenic microorganisms and they are protected by a large and highly specialized innate and adaptive mucosal immune system [1]. The adaptive humoral immune defense at mucosal surfaces is to a large extent mediated by secretory immunoglobulin A (SIgA), the predominant immunoglobulin class in human external secretion [2]. Adaptive humoral mucosal immune responses are mainly initiated in an inductive site (e.g., Peyer's patch in intestine). Sensitized mucosal immunocytes (e.g., IgA^+ ^B cells) then leave the inductive site, travel through the lymph, enter the circulation, and migrate to diffuse mucosal effector sites (e.g., lamina propria), where they differentiate into memory or effector cells (e.g., IgA-producing plasma cells) [3,4]. SIgA in saliva has been widely used as an indicator of mucosal immunity [5]. The salivary glands are the most important source of SIgA in the upper respiratory tract [6]. A lack of non-specific SIgA at the mucosal surface or the inability to produce specific SIgA can lead to an increased risk of infection [7].

Infectious diseases are one of the leading causes of mortality and significant morbidity in the elderly, who are at greater risk than the younger population [8]. Increasing age has been associated with a decline in humoral and cell-mediated immunity against newly encountered pathogens or vaccines [9-13], thus creating a need for countermeasures against age-related immune dysfunctions. SIgA secretion in saliva decreases with age [14-18], and may lead to an increased risk of respiratory infections. Therefore, improving or slowing the age-related decline of salivary SIgA may be beneficial for improving the health of the elderly.

Recently, many lactic acid bacteria have been reported to modulate specific/non-specific immune responses [19]. Certain species have been documented to enhance IgA secretion/concentration in the gut of mice [20], the feces of healthy children [21], and the saliva of infants [22]. However, no studies have been conducted on the augmentation of salivary SIgA secretion in the elderly. *Lactobacillus pentosus *strain b240 (b240) is an anaerobic non-sporulating gram-positive bacterium, originally isolated from fermented tea leaves [23]. Initially, on the basis of a carbohydrate-fermentation test and information from 16 S rRNA gene sequencing, this bacterium was identified as *Lactobacillus plantarum*. Recently, Bringel et al. [24] proposed its reclassification as *L. pentosus *on the basis of the recA gene sequence. Following this proposal, the gene sequence of b240 was analyzed in detail (data not shown) and formally classified as *L. pentosus*. In our laboratory, we have demonstrated b240 enhancement of IgA production from Peyer's patch cells in the mouse gut [25]. We have further determined that the oral intake of heat-killed b240 (corresponding to 2 × 10^9 ^or 2 × 10^10 ^CFU/day for 3 weeks) significantly elevates salivary IgA secretion rate and the amount of fecal IgA (but not significantly) in younger adults [26]. Taken together, these results indicate that b240 intake may increase salivary SIgA secretion via activation of the gut mucosal immune system. The objective of this study was to verify the hypothesis that oral intake of b240 is effective in enhancing salivary SIgA secretion in the elderly.

## Results

### Subject characteristics

The clinical features of the individuals who participated in this randomized control study at the initial (pre-intervention) and final (post-intervention) visits are shown in Table 1. There were no significant differences between the b240 and placebo groups with respect to age, height, weight, serum cortisol levels, saliva chromogranin A/total protein ratio, stress, total energy expenditure, and energy intake.

**Table 1:** Subject characteristics of the b240 and placebo groups before and after the 12-week study

	Pre-intervention		test	Post-intervention		test
				
	Placebo group	**b240 group**^***2***^	***P *****value**	Placebo group	**b240 group**^***2***^	***P *****value**
Number of subjects (male/female)	35 (9/26)	38 (10/28)				
Age (y)	71 ± 5	71 ± 5	0.846			
Height (cm)	155 ± 8	154 ± 6	0.497	-	-	
Body weight (kg)	55.3 ± 8.7	55.5 ± 6.8	0.916	-	-	
BMI (kg/m^2^)	22.9 ± 2.8	23.4 ± 2.5	0.447	-	-	
Serum cortisol (μg/dL)	12.0 ± 3.6	11.3 ± 3.6	0.403	11.3 ± 3.2	10.9 ± 4.6	0.721
Saliva chromogranin A/total protein ratio	7.7 ± 5.1	9.7 ± 7.5	0.186	9.1 ± 7.3	9.2 ± 6.0	0.920
POMS^*1 *^(points)						
Tension-Anxiety	48 ± 8	50 ± 10	0.289	48 ± 8	49 ± 9	0.751
Depression-Dejection	52 ± 10	53 ± 11	0.900	51 ± 10	51 ± 10	0.727
Anger-Hostility	51 ± 10	51 ± 8	0.999	49 ± 8	49 ± 9	0.796
Vigor	51 ± 7	53 ± 8	0.193	51 ± 9	53 ± 8	0.344
Fatigue	50 ± 9	52 ± 10	0.301	51 ± 10	52 ± 11	0.583
Confusion	51 ± 8	51 ± 11	0.963	50 ± 10	52 ± 9	0.516
Energy expenditure (kcal/day)	1900 ± 327	1939 ± 296	0.591	1959 ± 335	1969 ± 298	0.892
Energy intake (kcal/day)	2003 ± 525	1948 ± 620	0.685	2008 ± 607	1934 ± 653	0.618
Protein/Total energy intake (%)	16.9 ± 3.6	16.8 ± 2.7	0.883	16.8 ± 2.5	16.5 ± 2.8	0.644
Fat/Total energy intake (%)	28.6 ± 5.1	28.4 ± 5.1	0.881	28.7 ± 4.7	28.0 ± 5.4	0.568
Carbohydrate/Total energy intake (%)	51.2 ± 6.8	52.3 ± 5.8	0.444	51.5 ± 6.3	52.8 ± 5.7	0.385

Mean (SD); *P *value, by unpaired *t*-test.

*^1^*POMS, Profile of Mood States.

*^2^*4 × 10^9 ^cells of heat-killed *L. pentosus *strain b240 per day for 12 weeks.

### Overall changes in salivary variables

**Figure 1:**
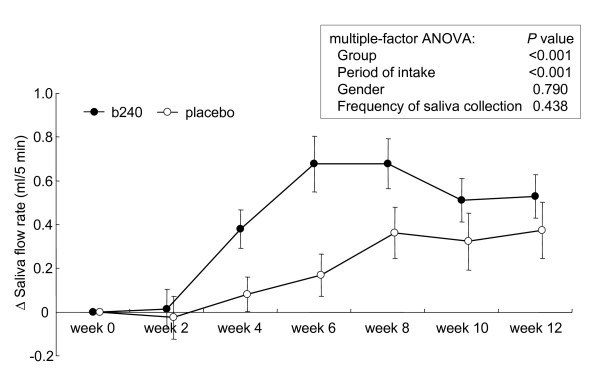
**Changes in saliva flow rate**. Changes in the saliva flow rate of subjects during the 12-week study with *L. pentosus *strain b240 (b240) beverage (closed circle) or placebo (open circle). Data are represented by mean ± SEM. The difference between the b240 and placebo groups was evaluated by multiple-factor ANOVA (factors: group, gender, period of intake, and frequency of saliva collection).

**Figure 2:**
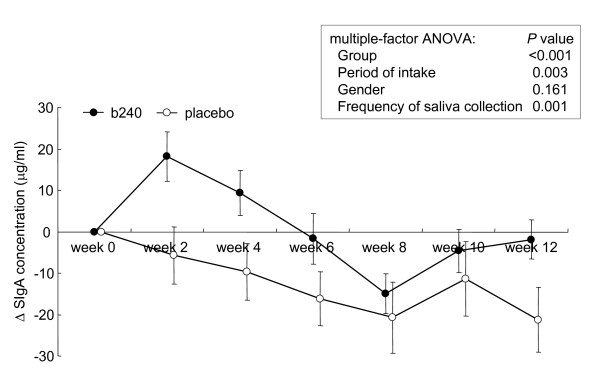
**Changes in salivary SIgA concentration**. Changes in the salivary SIgA concentration of subjects during the 12-week study with *L. pentosus *strain b240 (b240) beverage (closed circle) or placebo (open circle). Data are represented by mean ± SEM. The difference between the b240 and placebo groups was evaluated by multiple-factor ANOVA (factors: group, gender, period of intake, and frequency of saliva collection).

**Figure 3:**
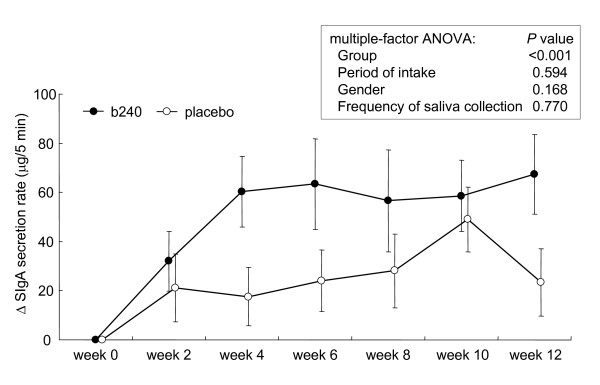
**Changes in salivary SIgA secretion rate**. Changes in the salivary SIgA secretion rate of subjects during the 12-week study with *L. pentosus *strain b240 (b240) beverage (closed circle) or placebo (open circle). Data are represented by mean ± SEM. The difference between the b240 and placebo groups was evaluated by multiple-factor ANOVA (factors: group, gender, period of intake, and frequency of saliva collection).

The mean absolute values for the 3 indices, salivary SIgA secretion rate, SIgA concentration, and saliva flow rate over the course of intervention are shown in Table 2. There were no significant differences between the b240 and placebo groups by statistical analysis of multiple-factor analysis of variance (ANOVA; factors: group, gender, period of intake, frequency of saliva collection). The salivary SIgA secretion rate at week 0 was subject to wide inter-individual variation (range, 87-674 μg/5 min; median, 296 μg/5 min). The averages of the individual changes in the saliva flow rate, SIgA concentration, and SIgA secretion rate over time (measurement at each visit minus individual baseline measurement) are shown in Figure 1, 2 and 3, respectively. The initial indices, saliva flow rate, SIgA concentration, and SIgA secretion rate were confirmed by the *t*-test as being statistically identical between the 2 groups. The average individual changes of saliva flow rate (Figure 1) and SIgA secretion rate (Figure 3) were increased in both groups. In contrast, average SIgA concentration decreased in the placebo group, while it increased in the b240 group until 4 weeks and returned to a prior level (Figure 2). The average individual changes of saliva flow rate were found to be significantly higher in the b240 group than in the placebo group at weeks 4 and 6 (*P *= 0.013 and *P *= 0.002, respectively). Likewise, the average SIgA concentrations were higher at weeks 2, 4, and 12 (*P *= 0.010, *P *= 0.030, and *P *= 0.037, respectively) and the average SIgA secretion rates were higher at weeks 4 and 12 (*P *= 0.024 and *P *= 0.042, respectively). Most importantly, the statistical multiple-factor ANOVA (factors: group, gender, period of intake, and frequency of saliva collection) revealed a significant elevation of all indices in the b240 group over those of the placebo group. The *P *values for group, gender, period of intake, and frequency of saliva collection period, respectively, are as follows: for saliva flow rate: <0.001, 0.790, <0.001, and 0.438; for SIgA concentration: <0.001, 0.161, 0.003, and 0.001; and for SIgA secretion rate: <0.001, 0.168, 0.594, and 0.770 (Figure 1, 2 and 3). In the b240 group, the mean SIgA secretion rate steadily increased by week 4 to 20% greater than at week 0, after which it remained stable until week 12.

**Table 2:** SIgA secretion rate, SIgA concentration, and saliva flow rate in the b240 and placebo groups

	Placebo group						
	
	Week 0	Week 2	Week 4	Week 6	Week 8	Week 10	Week 12
n	70	68	66	64	62	60	60
SIgA secretion rate^*2 *^(μg/5 min)	319 ± 16	337 ± 21	333 ± 20	343 ± 21	348 ± 23	363 ± 22	334 ± 20
SIgA concentration (μg/mL)	173 ± 13	170 ± 11	161 ± 12	159 ± 11	157 ± 11	165 ± 12	154 ± 11
Saliva flow rate (mL/5 min)	2.5 ± 0.2	2.4 ± 0.2	2.7 ± 0.2	2.7 ± 0.2	2.8 ± 0.2	2.8 ± 0.2	2.9 ± 0.2

	**b240 group^*1*^**						
	
	**Week 0**	**Week 2**	**Week 4**	**Week 6**	**Week 8**	**Week 10**	**Week 12**
**n**	76	75	70	72	62	70	66
	
SIgA secretion rate^*2 *^(μg/5 min)	297 ± 14	330 ± 20	357 ± 23	364 ± 27	366 ± 30	352 ± 23	359 ± 24
SIgA concentration (μg/mL)	147 ± 8	165 ± 10	156 ± 9	149 ± 11	134 ± 8	144 ± 8	149 ± 9
Saliva flow rate (mL/5 min)	2.3 ± 0.1	2.3 ± 0.2	2.6 ± 0.2	2.9 ± 0.2	3.0 ± 0.2	2.8 ± 0.2	2.7 ± 0.2

Mean ± SEM; n, number of saliva sample; SIgA; secretory immunoglobulin A.

*^1^*4 × 10^9 ^cells of heat-killed *L. pentosus *strain b240 per day for 12 weeks.

*^2^*SIgA secretion rate, which was calculated by multiplying SIgA concentration by saliva flow rate.

### Blood chemistry and adverse events

There were no significant differences in the blood chemistry profiles of the b240 and placebo groups as measured during pre-and post-intervention visits (Table 3). Accumulated incidences of adverse symptoms during the intervention did not differ significantly between groups (Table 4).

**Table 3:** Comparison of blood variables at pre- and post-intervention between the b240 and placebo groups

	Pre-intervention			Post-intervention		
				
Blood variable	Placebo group (n = 40)	b240 group*^1^* (n = 40)	*P *value	Placebo group (n = 40)	b240 group*^1^* (n = 40)	*P *value
Total protein (g/dL)	7.4 ± 0.4	7.2 ± 0.6	0.137	7.3 ± 0.4	7.2 ± 0.5	0.413
Albumin (g/dL)	4.4 ± 0.2	4.3 ± 0.2	0.115	4.4 ± 0.2	4.3 ± 0.3	0.381
Albumin/ globulin ratio	1.5 ± 0.2	1.5 ± 0.2	0.733	1.5 ± 0.2	1.5 ± 0.2	0.744
Total cholesterol (mg/dL)	221 ± 29	219 ± 35	0.722	221 ± 30	223 ± 37	0.814
Triglyceride (mg/dL)	105 ± 51	99 ± 53	0.631	101 ± 53	103 ± 50	0.895
Free fatty acid (mEq/L)	0.6 ± 0.2	0.6 ± 0.2	0.126	0.5 ± 0.2	0.5 ± 0.2	0.166
Fasting glucose (mg/dL)	95 ± 13	92 ± 12	0.355	95 ± 16	94 ± 16	0.863
Hemoglobin A_1c _(%)	5.2 ± 0.4	5.3 ± 0.4	0.536	5.3 ± 0.4	5.4 ± 0.4	0.663
Aspartate amino-transferase (U/L)	24.5 ± 7.2	25.7 ± 9.1	0.506	23.1 ± 8.6	23.6 ± 9.5	0.825
Alanine amino-transferase (U/L)	18.9 ± 7.9	20.4 ± 7.5	0.387	18.1 ± 10.1	18.3 ±7.2	0.939
γ-Glutamyl transpeptidase (U/L)	27.8 ± 18.3	28.7 ± 26.2	0.863	27.1 ± 21.4	29.4 ± 34.9	0.724
Alkaline phosphatase (U/L)	258 ± 74	265 ± 131	0.763	245 ± 67	262 ± 143	0.506
Lactate dehydrogenase (U/L)	216 ± 51	205 ± 62	0.386	194 ± 25	184 ± 27	0.113
Choline- esterase (U/L)	340 ± 50	328 ± 47	0.258	325 ± 48	319 ± 48	0.619
Total bilirubin (mg/dL)	0.6 ± 0.2	0.7 ± 0.3	0.058	0.7 ± 0.2	0.7 ± 0.3	0.315
Direct bilirubin (mg/dL)	0.2 ± 0.0	0.2 ± 0.1	0.122	0.2 ± 0.1	0.2 ± 0.1	0.752
Indirect bilirubin (mg/dL)	0.5 ± 0.1	0.5 ± 0.2	0.078	0.5 ± 0.2	0.6 ± 0.2	0.241
Urea nitrogen (mg/dL)	14.8 ± 3.6	15.1 ± 3.6	0.695	15.7 ± 4.5	15.9 ± 3.5	0.846
Uric acid (mg/dL)	4.8 ± 1.0	4.9 ± 1.0	0.771	5.0 ± 1.0	5.1 ± 1.2	0.983
Creatinine (mg/dL)	0.7 ± 0.2	0.7 ± 0.1	0.894	0.7 ± 0.2	0.7 ± 0.1	0.604
Sodium (mEq/L)	141 ± 2	141 ± 1	0.564	141 ± 2	141 ± 2	0.944
Chloride (mEq/L)	104 ± 2	103 ± 2	0.348	103 ± 2	104 ± 3	0.852
Potassium (mEq/L)	4.8 ± 0.5	4.9 ± 0.5	0.508	4.5 ± 0.5	4.4 ± 0.4	0.436
Calcium (mg/L)	9.3 ± 0.3	9.2 ± 0.4	0.238	9.3 ± 0.3	9.2 ± 0.4	0.293
Inorganic phosphorus (mg/dL)	3.4 ± 0.3	3.4 ± 0.5	0.955	3.4 ± 0.3	3.3 ± 0.5	0.386
Red blood cells (×10^4^/μL)	439 ± 33	443 ± 32	0.643	433 ± 32	437 ± 28	0.596
White blood cells (/μL)	5493 ± 1270	5420 ± 1205	0.794	5258 ± 1362	5058 ± 1089	0.470
Platelet (×10^4^/μL)	22.3 ± 4.4	21.9 ± 5.2	0.693	22.0 ± 4.1	21.0 ± 5.0	0.349
Hemoglobin (g/dL)	13.9 ± 0.9	13.9 ± 0.9	0.933	13.5 ± 0.9	13.5 ± 0.8	0.880
Hematocrit (%)	43.5 ± 2.7	43.6 ± 2.5	0.942	43.4 ± 2.9	43.6 ± 2.3	0.779

Mean (SD); n, number of subject; *P *value, by unpaired *t*-test.

*^1^*4 × 10^9 ^cells of heat-killed *L. pentosus *strain b240 per day for 12 weeks.

**Table 4:** Accumulated incidents of adverse symptoms reported by the subjects during the intervention period

Symptoms	Placebo group (n = 40)	**b240 group**^***1***^ (n = 40)	***P *****value**
Common cold	4 (10.0%)	2 (5.0%)	0.396
Headache	1 (2.5%)	2 (5.0%)	0.556
Anorexia	2 (5.0%)	0 (0.0%)	0.152
Nasal discharge	4 (10.0%)	7 (17.5%)	0.330
Abdominal distension	0 (0.0%)	1 (2.5%)	0.314
Abdominal pain	0 (0.0%)	1 (2.5%)	0.314

Data are represented by number of symptoms (incidence of symptoms) during the intervention period. *P *value, by the chi-square test.

*^1^*4 × 10^9 ^cells of heat-killed *L. pentosus *strain b240 per day for 12 weeks.

## Discussion

The major finding of this study was that, compared to placebo, the oral intake of *L. pentosus *strain b240 (4 × 10^9 ^cells) for 12 weeks resulted in higher SIgA secretion rate in the elderly. This is the first report to show that salivary SIgA in the elderly can be augmented by supplementation with lactic acid bacteria.

The elevated SIgA secretion rate in the b240 group reached a plateau at week 4, suggesting that b240 intake continuously elevates the rate of SIgA secretion during the first month. Thereafter, the SIgA secretion rate remained stable from week 4 until week 12, suggesting that continuous daily intake of b240 may be efficacious for the maintenance of increased of salivary SIgA in the elderly.

How did oral intake of b240 lead to an augmentation of salivary SIgA? Within the mucosal immune system, there is a significant degree of compartmentalization linking specific mucosal inductive sites with particular effector sites (e.g., the gut with mammary and salivary glands), which is partially due to the differential expression of chemokines, integrins and cytokines among mucosal tissues [3,4]. In line with this study, the oral intake of polybacterial immunomodulator Dentavax, containing killed *Lactobacillus acidophilus*, has been reported to induce a strain-specific IgA response in the saliva of healthy adults [27]. Likewise, the oral intake of fermented milk containing *L. casei *DN-114 001 has been shown to induce non-specific total SIgA in infant saliva [22]. One plausible hypothesis is that the oral intake of b240 stimulates the gut mucosal immune system to enhance secretion of SIgA in saliva.

Saliva is an easily retrievable sample material and can be collected non-invasively. Thus, salivary SIgA has been widely used to assess the status of the mucosal immune system. There are 2 main outcome measures for evaluation of salivary SIgA: secretion rate and concentration. In this study, we focused on the SIgA secretion rate because it represents the actual amount of SIgA available on the mucosal surfaces for protection against pathogens [28]. Furthermore, Fahlman et al. [29] argued that, of all the various methods commonly employed for SIgA assessment, the SIgA secretion rate is the most useful clinical biomarker for predicting the incidence of upper respiratory tract infections (URTIs) using 8 separate stepwise multiple regression analyses. According to two longitudinal (about 1-year) studies of elite athletes, decreased levels of salivary SIgA secretion were associated with an increased risk for URTI; in these studies, mean SIgA secretion relative to baseline was reduced by 25-28% over the course of observation [29,30]. In the present study, b240 intake for 12 weeks produced a 20% increase in SIgA secretion rate relative to baseline in a group of elderly participants, thereby indicating potential clinical relevance.

In the present study, we used heat-killed b240. Heat-killed bacteria were chosen over viable bacteria for two reasons. First, our previous animal experiments have shown that mouse Peyer's patch cells, when treated with viable or heat-killed b240 for 21 days produced approximately the same level of IgA [25]. Second, our previous human study demonstrated that the oral intake of heat-killed b240 enhanced salivary IgA secretion in younger adults [26]. Several earlier studies support the view that viability is not necessary to achieve probiotic immunomodulatory effects [31,32]. However, further studies are needed to compare the effectiveness of heat-killed and viable b240 in terms of clinical application in the elderly.

This study has several methodological strengths. First, minimization of intra-individual variations in SIgA secretion rate was achieved by selecting non-smoking subjects and by collecting saliva samples throughout the intervention period at the same time of the day, when the salivary SIgA secretion rate is stable [33]. The salivary SIgA secretion rate generally shows wide inter- and intra-individual variation [16,17,33-36]. Our procedures enabled us to monitor the resting state of salivary SIgA secretion while minimizing potential errors. Second, we adopted the suction method for collecting saliva samples. There have been many studies on salivary SIgA that have used different sampling methods [37-40]. Michishige et al. [37] compared 3 saliva collection methods--spitting, suction, and swab methods--and concluded that the suction method is ideal for obtaining a stable saliva profile. Prior to this study, we compared the salivary SIgA secretion rate in younger adults and the elderly using the suction and swab methods. The suction method provided more stable results than the swab method [unpublished results]. Third, we successfully controlled the measured (age, gender, stress, energy intake, energy expenditure, etc.) and non-measured confounders, which could affect study results.

During the intervention, the saliva flow rate per 5 min (mL/5 min) was elevated in both groups. Saliva flow rate has been reported to show seasonal variations [41,42]. Takaoka et al. [41] noted that unstimulated whole saliva volume increases during spring, reaches a plateau in summer, and decreases from autumn to winter. Since the present study was performed from February to May, this seasonal variation may explain why the saliva volume increased during the trial. Compared with the placebo group, the b240 group had a greater incremental increase in saliva flow rate, which would be expected as a result of the stimulatory effect of b240. It has been reported that the SIgA concentration in saliva is negatively correlated with the saliva flow rate [5], possibly explaining why the placebo group exhibited a decrease in SIgA levels during the trial. In the b240 group, despite the fact that the saliva flow rate increased relative to that of the placebo group, the SIgA concentration increased until week 4 relative to its initial value and returned to a prior level. This could be explained by the enhanced seasonal salivary secretion effect. Taken together, these results indicated that the intake of b240 elevates the salivary SIgA secretion rate by stimulating not only the saliva flow rate but also SIgA production.

There were no significant differences between the 2 groups; blood profiles before and after the study, indicating that the study had no consistent effect on the blood components we examined. Furthermore, the number of incidents of unfavorable changes in the blood did not differ between the 2 groups (data not shown). A review of illness records over the intervention period revealed no significant difference in adverse events between the 2 groups; this is consistent with our previous results [26]. However, future studies using a larger sample size are needed to confirm the safety of oral intake of b240.

This study had several limitations. First, we excluded inappropriate saliva samples that met pre-determined exclusion criteria for data analysis. This procedure resulted in different saliva sample sizes in the b240 and placebo groups. However, exclusion/missing data occurred less often in the b240 group than in the placebo group, and an alternative analysis (intention-to-treat approach) using all available samples, including potentially inappropriate samples, generated almost identical results (data not shown). Second, this study only included individuals who had never been smokers. The results of this study therefore cannot be automatically generalized to be applicable to smokers. Third, a comparison with younger counterparts is lacking in this study. In our previous report, however, we demonstrated the beneficial effect of b240 on salivary IgA in young adults [26], in which we used the same dosage of b240 (2 × 10^9 ^CFU/day), suggesting that all adults respond in a similar fashion to oral b240 intake. However, a direct comparison between young and older subjects may provided further information on the b240 mechanism of action, e.g., normalization of the SIgA response in the elderly or vice versa and the need to increase dosage and time of administration for attaining normality.

## Conclusions

This study has shown that b240 intervention increases SIgA secretion in the saliva of elderly individuals. Improved clinical endpoints as a product of mucosal immunity may be achieved by increasing SIgA secretion. It is now important to determine whether enhanced salivary SIgA secretion could lead to increased protection against infectious diseases such as URTI in the elderly.

## Methods

### Subjects

**Figure 4:**
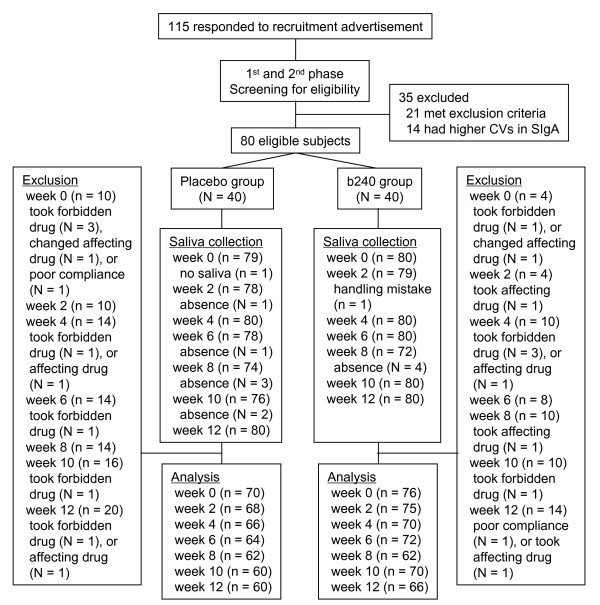
**Flow of subjects and saliva samples through the trial**. N, number of subjects; n, number of saliva samples; CV, coefficient of variation. Saliva samples were collected twice per person every 2 weeks during the 12 week period. "Affecting drug" means that the drug, such as an antihistamine, potentially affects saliva secretion. "Absence" means that the subject could not participate on the day of saliva collection due to miscellaneous reasons. "No saliva" means that the subject was present but failed to provide a saliva sample.

The research protocol was approved by the Ethics Committee of Tokyo Metropolitan Institute of Gerontology (TMIG) (November 26, 2007). The study was conducted in accordance with the Helsinki Declaration. The subjects were recruited via a local advertisement for residents aged 65 years and over and residing in Itabashi Ward, Tokyo. Before giving consent, the subjects were given a detailed explanation of the purposes of the study and potential risks of participation. A total of 115 individuals participated in the first-phase screening test (see Assessment). The initial exclusion criteria were as follows: (1) current or former smoker; (2) vigorous (more than 6 metabolic equivalents) exerciser; (3) showing non-standard results in blood chemistry, blood pressure, and pulse; (4) patients with hepatitis, cancer, inflammatory bowel diseases, rheumatoid arthritis, and other diseases affecting the digestive tract and immune system; (5) patients with periodontitis and/or hemorrhagic stomatitis; (6) patients taking antibiotics; (7) patients taking antiflatulents, antidiarrheic drugs, steroids, immunosuppressive drugs, and other drugs related to activation and suppression of the digestive and immune systems; (8) patients taking drugs potentially affecting saliva secretion; and (9) patients declared ineligible for participation by a medical doctor. Throughout the test, 21 out of the 115 participants were found to meet at least 1 of the exclusion criteria and were excluded. The remaining 94 individuals underwent a second-phase screening test in which they visited the study site twice on 2 different days in a week and provided a total of 4 saliva samples (2 samples per day × 2 days); the samples were used to determine SIgA levels. The saliva collection and SIgA determination method was identical to the methods described below. According to the coefficient of variance (CV) in the SIgA secretion rate of 4 saliva samples, we further selected 80 individuals who showed relatively low CVs. They included the subjects selected for the following randomized controlled trial (RCT) (Figure 4).

### Protocol of the randomized control trial

The experiment was conducted from February to May 2008 as a randomized, placebo-controlled, double-blind trial. 80 eligible subjects were randomly assigned to 1 of 2 groups: the b240 group (n = 40) and placebo group (n = 40), matched for age and gender. The participants were instructed to consume a beverage containing b240 or placebo daily at breakfast time for 12 weeks. On the day of saliva collection, they were given a beverage immediately after saliva collection. Saliva samples were collected using the suction method at the same time of day before (week 0) and every 2 weeks (weeks 2, 4, 6, 8, 10, and 12) after study initiation. The subjects were also instructed to record in a diary any symptoms experienced and/or medications taken during the 12-week intervention. This information was provided for the evaluation of safety and eligibility for saliva sample procedures.

### b240 and placebo beverage

A trial beverage containing 4 × 10^9 ^cells of heat-killed b240 was prepared in 125 mL of sterile water as follows: After cultivating b240 in carrot juice and performing repeated washing, viable cells were counted using the colony counting method. The b240 cells were sterilized in an autoclave for 15 min, diluted with sterile water to a final concentration of 2 × 10^9 ^CFU/125 mL based on viable cell counts. Then, total cell counts were determined as 4 × 10^9 ^cells/125 mL using flow cytometry. Aliquots of 125 mL were placed in paper cups. A placebo beverage (125 mL of sterile water) was similarly prepared. Both beverages had identical taste. The identity of the beverage consumed by each individual subject was double-blinded until the end of the study.

### Assessment

In the first phase of screening, all participants (n = 115) were assessed with respect to their physical characteristics [height, weight, body mass index (BMI)], blood pressure, urine profile, oral health, functional capacity, and physical fitness. For this purpose, the individuals were interviewed by trained personnel on their medical history, medications, TMIG-Index of Competence [43], and Motor Fitness Scale [44]. All subjects underwent urinalysis and blood tests and were examined for periodontitis and hemorrhagic stomatitis by a dental hygienist. We used this information as pre-intervention data for the 80 RCT-eligible subjects. Additionally, the 80 subjects were examined for stress and mood, nutritional intake, and physical activities by answering the Profile of Mood States (POMS) questionnaire [45], the Brief Dietary Habit Questionnaire [46,47], and the Japan Arteriosclerosis Longitudinal Study Physical Activity Questionnaire [48]. Chromogranin A (CgA) in saliva was also measured.

After the intervention, those who completed the RCT (n = 80) were assessed again for all the items mentioned above except for physical characteristics, blood pressure, urine profile, oral health, functional capacity, and physical fitness.

From the subjects' diaries, we collected information regarding protocol compliance, occurrence of adverse symptoms, and medications used throughout the intervention period.

### Blood tests and chromogranin A in saliva

Blood was tested for circulating cell counts (red blood cells, white blood cells, and platelets), hemoglobin, hematocrit, serum total protein, albumin, albumin/globulin ratio, total cholesterol, triglyceride, free fatty acid, fasting glucose, hemoglobin A1c, aspartate aminotransferase, alanine aminotransferase, γ-glutamyl transpeptidase, alkaline phosphatase, lactate dehydrogenase, choline esterase, total bilirubin, direct and indirect bilirubin, urea nitrogen, uric acid, creatinine, sodium, chloride, potassium, calcium, inorganic phosphorus, and cortisol by standardized procedures at the Special Reference Laboratory Inc., Tokyo, Japan. CgA in saliva was measured using the Human Chromogranin A EIA Kit (Yanaihara Inst., Shizuoka, Japan), and total protein in saliva was measured using a protein staining assay (Bio-Rad Japan, Tokyo, Japan). CgA was evaluated by the CgA/total protein ratio.

### Saliva collection and SIgA detection

Saliva samples were collected every 2 weeks during the 12-week period, according to the method described by Michishige et al. [37]. Subjects were asked to avoid alcoholic beverages and to fast overnight before the test; they gathered at a test site, consumed an identical breakfast (250 kcal in energy), and rested in a seated position until the initiation of saliva collection. 5 minutes before the sample collection, all the subjects rinsed their mouths with distilled water 3 times, swallowed the whole saliva, and fixed a plastic suction tube (TOP Co., Tokyo, Japan) in their mouth to allow aspiration of fresh saliva for 5 min (SeaStar Co., Tokyo, Japan). After a 15-min rest, saliva collection was repeated to yield 2 samples per individual. The amount of saliva in grams was converted to milliliters assuming the density of saliva to be 1 g/mL. After measurement of the sample weight, the saliva samples were frozen at -80°C and stored until further use. SIgA concentration was measured by an enzyme-linked immunosorbent assay using a commercial kit (Salivary Secretory IgA Indirect Enzyme Immunoassay Kit; Salimetrics, PA, USA). The intra-assay CV for SIgA concentration with a higher and a lower value was 2.3% and 2.8%, respectively. The inter-assay CV for SIgA concentration with a higher and a lower value was 3.8% and 5.1%, respectively. Data were expressed in 3 forms: (1) saliva flow rate per 5 min (mL/5 min), (2) SIgA concentration (μg/mL), and (3) SIgA secretion rate per 5 min (μg/5 min); the SIgA secretion rate was calculated by multiplying the SIgA concentration (μg/mL) by the saliva flow rate per 5 min (mL/5 min).

### Assessment of safety

We compared the accumulated incidents of adverse symptoms occurring during the intervention and blood profiles at pre- and post-intervention in the b240 and placebo groups.

### Statistical analyses

Before subject allocation, potentially inappropriate saliva samples were excluded from the analysis on the basis of the following exclusion/inclusion criteria: all samples for a non-compliant subject and for 2 subjects who changed antihistamine medications during the study period, immediate samples for a subject who had several alcoholic beverages in the evening prior to collection, all samples thereafter for subjects who took antibiotics (n = 8), steroids (n = 1), and drugs related to the activation of digestive tract function (n = 2), and immediate samples for 5 subjects who temporarily took antihistamines shortly before saliva collection. These procedures yielded a final sample size of 66 (week 12) for the b240 group (week 0 sample size, 76), and 60 (week 12) for the placebo group (week 0 sample size, 70) (2 saliva samples were collected per person per day; Figure 4).

Salivary SIgA concentration, saliva flow rate, and SIgA secretion rate were analyzed separately. The differences between the b240 and placebo groups for each variable were analyzed by multiple-factor ANOVA (factors: group, gender, period of intake, and frequency of saliva collection). The comparison between the b240 and placebo groups with respect to age, height, weight, BMI, serum cortisol, CgA/total protein ratio, stress assessment, nutritional intake, and daily physical activities were analyzed by the unpaired *t*-test. The unpaired *t*-test was applied to the comparison of blood variables pre- and post-intervention and the chi-squared test was applied to compare the accumulated incidents of adverse symptoms between groups. A two-tailed *P-*value <0.05 was accepted as significant for all tests. Data were analyzed using SAS software (R8.1 or R9.1, SAS Institute, Japan).

## List of Abbreviations

IgA: Immunoglobulin A; SIgA: secretory immunoglobulin A; ANOVA: analysis of variance; URTI: upper respiratory tract infection; TMIG: Tokyo Metropolitan Institute of Gerontology; CV: coefficient of variance; RCT: randomized controlled trial; BMI: body mass index; POMS: Profile of Mood States; CgA: chromogranin A.

## Competing interests

The authors declare that they have no competing interests.

## Authors' contributions

YK directed all aspects of the study and contributed to the preparation of the manuscript. SS designed experiments, collected data, and contributed to the preparation of the manuscript. HO, MT, and NK designed experiments and provided helpful advice. PHMC contributed to the preparation of the manuscript. KO collected data and contributed to the preparation of the manuscript. HY, TF, and YF contributed to the collection of data. KK performed the statistical analyses. All authors read and approved the manuscript.
